# Linking regional variation of epibiotic bacterial diversity and trophic ecology in a new species of Kiwaidae (Decapoda, Anomura) from East Scotia Ridge (Antarctica) hydrothermal vents

**DOI:** 10.1002/mbo3.227

**Published:** 2014-12-16

**Authors:** Katrin Zwirglmaier, William D K Reid, Jane Heywood, Christopher J Sweeting, Benjamin D Wigham, Nicholas V C Polunin, Jeff A Hawkes, Douglas P Connelly, David Pearce, Katrin Linse

**Affiliations:** 1 British Antarctic SurveyHigh Cross, Madingley Road, Cambridge, CB3 0ET, United Kingdom; 2 School of Marine Science and Technology, Newcastle UniversityNewcastle Upon Tyne, NE1 7RU, United Kingdom; 3 Dove Marine Laboratory, School of Marine Science and Technology, Newcastle UniversityCullercoats, NE30 4PZ, United Kingdom; 4 National Oceanography CentreEuropean Way, Southampton, SO14 3ZH, United Kingdom; 5 Faculty of Health and Life Sciences, University of NorthumbriaEllison Building, Newcastle Upon Tyne, NE1 8ST, United Kingdom; 6 University Centre in SvalbardP.O. Box 156, N-9171, Longyearbyen, Norway

**Keywords:** East Scotia Ridge, epibionts, hydrothermal vent, Kiwa sp., microbial diversity, stable isotopes

## Abstract

We analyzed the diversity of bacterial epibionts and trophic ecology of a new species of *Kiwa* yeti crab discovered at two hydrothermal vent fields (E2 and E9) on the East Scotia Ridge (ESR) in the Southern Ocean using a combination of 454 pyrosequencing, Sanger sequencing, and stable isotope analysis. The *Kiwa* epibiont communities were dominated by *Epsilon*- and *Gammaproteobacteria*. About 454 sequencing of the epibionts on 15 individual *Kiwa* specimen revealed large regional differences between the two hydrothermal vent fields: at E2, the bacterial community on the *Kiwa* ventral setae was dominated (up to 75%) by *Gammaproteobacteria*, whereas at E9 *Epsilonproteobacteria* dominated (up to 98%). Carbon stable isotope analysis of both *Kiwa* and the bacterial epibionts also showed distinct differences between E2 and E9 in mean and variability. Both stable isotope and sequence data suggest a dominance of different carbon fixation pathways of the epibiont communities at the two vent fields. At E2, epibionts were putatively fixing carbon via the Calvin-Benson-Bassham and reverse tricarboxylic acid cycle, while at E9 the reverse tricarboxylic acid cycle dominated. Co-varying epibiont diversity and isotope values at E2 and E9 also present further support for the hypothesis that epibionts serve as a food source for *Kiwa*.

## Introduction

Hydrothermal vents are highly productive ecosystems, where primary production occurs through chemoautotrophic microbial production. They support dense communities of metazoans, some of which are in endo- or episymbiotic relationships with microbes. The spatial distribution of organisms within these habitats is governed by temperature and composition of chemically reduced fluids emitted from the seafloor as well as topology and substrate type (Cuvelier et al. 2009; Takai et al. 2009; Podowski et al. 2010; Flores et al. 2011; Petersen et al. 2011; Beinart et al. 2012). Metazoan communities at hydrothermal vents vary globally and a series of biogeographical provinces has been proposed (Moalic et al. 2012; Rogers et al. 2012). In areas closest to high temperature venting, alvinellid polychaetes (North Pacific), alvinochoncid gastropods (western Pacific), rimicarid shrimp (Atlantic Ocean), and the decapod *Shinkaia crosnieri* (western Pacific) are the dominant metazoan fauna in their respective biogeographical provinces (Tunnicliffe et al. 2003; Moalic et al. 2012). All these species are known to be in symbiotic relationships with either endo- or epibiotic bacteria.

**Figure 1:**
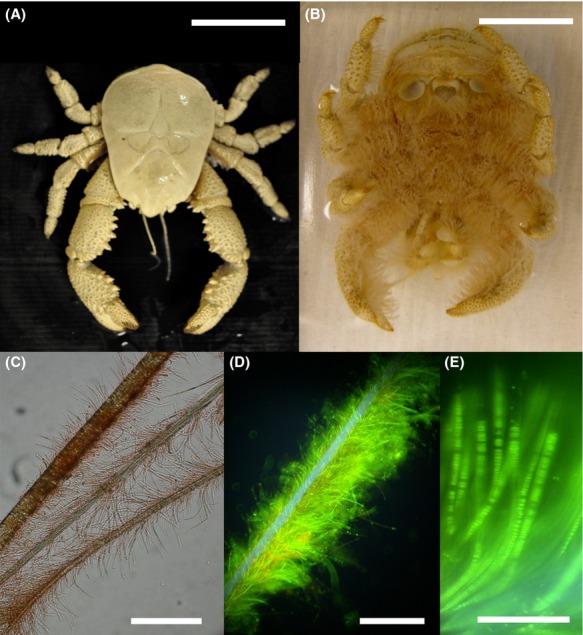
(A) *Kiwa* sp. nov. East Scotia Ridge (ESR), (B) ventral view, showing the setae covering the ventral side. (C) individual setae covered with epibiotic bacteria, (D and E) SYBR green staining of epibiotic bacteria. Scale bars: (A, B) – 5 cm, (C, D) – 100 *μ*m, (E) – 50 *μ*m.

In 2010, the first hydrothermal vent fields south of the polar front were discovered on the East Scotia Ridge (ESR) (Rogers et al. 2012). The vent fields are situated ca. 440 km apart on segments E2 and E9 of the ESR at a similar depth, E2 at ca. 2600 m and E9 at ca. 2400 m. Both have a number of actively venting black smoker chimneys, where temperatures of up to 353°C (E2) and 383°C (E9) have been measured (Rogers et al. 2012). The dominant fauna at both sites is a new species of anomuran crab (Fig.1A and B). Morphological and molecular analyses identified the anomuran crab as a new species of yeti crab within the genus *Kiwa* (Roterman et al. 2013), hereafter referred to as *Kiwa* sp. nov. ESR, which has ventral setae covered in filamentous epibiotic bacteria (Rogers et al. 2012). *Kiwa* sp. nov. ESR is found in densities of up to 4000 individuals m^−2^ (Marsh et al. 2012), much higher than the other two known Kiwaids, *Kiwa hirsuta* (Macpherson et al. 2005), found at Pacific-Antarctic Ridge hydrothermal vents, and *Kiwa puravida* (Thurber et al. 2011), found at cold methane seeps off the Coast of Costa Rica. Given the high abundance of species like *Kiwa* sp. nov. ESR (Marsh et al. 2012), *Rimicaris exoculata* (>1120 ind. m^−2^, Copley et al. 2007) and *S. crosnieri* (>560 ind. m^−2^, Tokeshi 2011) the contribution of epibiont communities to the overall biological production of hydrothermal vents food webs is likely to be high.

Chemolithoautotrophic bacteria can fix carbon via different pathways, which are largely conserved within a phylogenetic group (reviewed by Hügler and Sievert 2011). Within the *Epsilonproteobacteria*, all autotrophic members are believed to use the reductive tricarboxylic acid (rTCA) cycle (Campbell and Cary 2004; Hügler et al. 2005; Takai et al. 2005), whereas the Calvin-Benson-Bassham (CBB) cycle is typical for autotrophic *Gammaproteobacteria* (Hügler and Sievert 2011 and references therein). Differences in the carbon fixation pathways are reflected in the stable carbon isotope (*δ*^13^C) signatures of the microbial primary producers resulting in *δ*^13^C values less than -20‰ and greater than −16‰ for organic carbon fixed via CBB and rTCA cycle, respectively (Hügler and Sievert 2011). As a result of minimal trophic discrimination in *δ*^13^C between food source and consumer, a metazoan consumer's *δ*^13^C values reflect those of their carbon source (Hügler and Sievert 2011). Spatial variability in *δ*^13^C can therefore reveal the relative importance of different food sources within and among locations when individuals consume food sources which have widely different *δ*^13^C values (Bearhop et al. 2004). The phylogenetic composition of the epibiont community, that is, for example, dominated by either *Gammaproteobacteria* or *Epsilonproteobacteria*, in combination with the *δ*^13^C values can be an indication of the carbon fixation pathway utilized by epibionts, and thus the relative contribution of either the CBB or rTCA cycles to sustaining the metazoan consumer.

In order to investigate epibiont-*Kiwa* interactions, vent fluids, epibionts, and *Kiwa* sp. nov. ESR were collected concurrently at various sites within the E2 and E9 vent fields. Preliminary chemical and physical analysis has revealed that the vent fields have contrasting vent fluid end-member chemistries and there are also detectable differences within E9 between the northern (E9N) and southern (E9S) parts of the vent field (Rogers et al. 2012). The major aims of this study were to: (1) examine epibiont diversity of the newly discovered *Kiwa* sp. nov. ESR at E2 and E9; (2) compare the *Kiwa* sp. nov. ESR epibiont community to that harbored by other hydrothermal decapods, which occupy a similar ecological niche; (3) examine the stable isotope signature of *Kiwa* sp. nov. ESR to assess whether the degree of isotopic variability was consistent with epibiont diversity.

## Experimental Procedures

### Sampling and sample preparation

Samples were collected during cruise JC42 on board the RRS James Cook in January–February 2010 using the remotely operated vehicle (ROV) Isis. Specimen were collected at various sites (as listed in Table2 and Table S2) at the E2 and E9 vent fields in the ESR. For DNA extraction and sequencing, the *Kiwa* sp. nov. ESR specimen were either fixed in 100% ethanol and then stored at −80°C or frozen directly at −80°C until used. Carapace length of specimens (CL) was measured from the base of the rostrum to the posterior-lateral margin of the carapace.

### Microscope analysis

For SYBR green staining (Fig.1D and E), epibiont-covered setae of an ethanol-fixed *Kiwa* sp. nov. ESR were cut off with sterilized scissors and stained with a 200× solution of SYBR Green I (Sigma, St. Louis, MO) for 1 h at room temperature in the dark. The setae were washed with ddH_2_O and then analyzed with an epifluorescence microscope.

### DNA extraction

A patch of setae covered with epibiotic bacteria was cut off from the ventral side of the *Kiwa* sp. nov. ESR with sterilized scissors and DNA was extracted using a phenol/chloroform protocol. See supplementary data S1.

### PCR, cloning and Sanger sequencing

The 16S rRNA gene was amplified with the universal bacterial primers 1492R (Stackebrand and Liesack 1993) and 27F (Lane 1991). The PCR conditions were 94°C, 3 min, followed by 30 cycles of 94°C, 45 sec, 50°C, 30 sec, 72°C, 90 sec, and a final extension of 5 min at 72°C, using MyTaq polymerase (Bioline, Cambridge, UK). PCR products were cloned using TOPO TA cloning (Invitrogen/Life Technologies, Carlsbad, CA, USA) and sequenced bi-directionally (LGC Genomics, Berlin, Germany). Sequences have been submitted to Genbank (accession numbers KF438845–KF439049). Clone libraries for Sanger sequencing were generated from the epibionts of one E2 *Kiwa* sp. nov. ESR collected at Crab City and one E9 *Kiwa* sp. nov. ESR collected at Black and White.

### 454 sequencing

Primers 786Fm (Roesch et al. 2007) and 1194R (Maeda et al. 2003) were used to amplify part of the 16S rRNA gene. PCR conditions were 94°C, 3 min, followed by 30 cycles of 94°C, 45 sec, 50°C, 30 sec, 72°C, 60 sec, and a final extension of 5 min at 72°C using MyTaq polymerase (Bioline, UK Cambridge). Primer 786Fm was coupled with the fusion primer A plus a barcode to allow multiplexing, while primer 1194R was coupled with fusion primer B of the LibL chemistry. Sequencing was done from the 786R end, that is, sequences start just before V5 region. Sequencing was done by LGC Genomics (Berlin, Germany) on a Roche454 FLX Titanium sequencer 454 Life Sciences, Branford, CT. Sequence data have been submitted to Genbank, Biosample accession number SAMN02189919.

### Sequence analysis

Sanger sequences were quality checked, assembled and trimmed with Geneious (www.geneious.com) and further analyzed with arb (Ludwig et al. 2004). Principal coordinate analysis was done with Fast unifrac (Hamady et al. 2009), based on a maximum likelihood tree generated in arb. Only sequences >1300 bp were used.

454 sequence data were analyzed with mothur v18.0 (Schloss et al. 2009) and arb. For further details see supplementary data S1.

The P (phylogenetic) test (Martin 2002) was used to test for significant differences between the epibiont communities from different sampling sites. The *P*-test uses a phylogenetic tree of the sample sequences and a parsimony approach to estimate the number of sequence changes necessary to explain the different distribution of sequences between samples. This tool is incorporated in the Fast unifrac website.

### Stable isotope analysis

Muscle was removed from the chelipeds of *Kiwa* sp. nov. ESR, frozen at −80°C before being freeze dried and ground to a homogeneous powder for carbon and nitrogen SIA. Approximately 0.7 mg of powder was weighed into tin capsules and isotopic ratios were then measured by continuous-flow isotope ratio mass spectrometry using a Costech Elemental Analyzer interfaced with Thermo Finnigan Delta Plus XP (Natural Environment Research Council, Life Sciences Mass Spectrometry Facility, SUERC, East Kilbride, UK). Two laboratory standards were analyzed every ten samples in each analytical sequence. These alternated between paired alanine standards, of differing *δ*^13^C and *δ*^15^N, and an internal laboratory gelatin standard. Stable isotope ratios were expressed in delta (*δ*) notation as parts per mil (‰). All internal standards are traceable to the following international standards v-PDB (Pee Dee Belemnite) and AIR (atmospheric nitrogen). An external reference material of white fish muscle was also analyzed (*δ*^13^C, *n* = 24, −18.94‰ ± SD 0.09; *δ*^15^N, *n* = 24, 13.11‰ ± SD 0.38).

### Isotope data analysis

*Kiwa* sp. nov. ESR isotopic niches were examined using the dispersion of *δ*^13^C and *δ*^15^N values in *xy*-space by calculating sample size corrected standard ellipse areas (SEAc) (Jackson et al. 2011) using the SIAR package (Parnell et al. 2010) implemented in the R statistical package version 3.0.1. See Jackson et al. (2011) and Data S1 for more details.

The relationships between *δ*^13^C and CL were analyzed by linear regression. Regression diagnostics were examined to assess normality (qq-plots) and homogeneity of variance (standardized residuals vs. fitted values).

### Chemistry

Samples were taken and processed as described in Rogers et al. 2012 and James et al. 2014. Briefly, samples were taken by ROV using titanium major samplers linked to an inductively coupled temperature probe, and chloride and time sensitive parameters such as H_2_S and gases were fixed and analyzed on board. Major cations were analyzed by inductively coupled plasma – optical emission spectroscopy (ICP-OES) at the National Oceanography Centre Southampton after acidification, storage at 4°C and dilution. Results for each analyte from each vent chimney were corrected for seawater mixing by extrapolating to zero magnesium as is the convention, except for pH where the lowest measured result is reported.

## Results

### Physical and chemical properties of the vent fluids

The maximum temperatures of the vent fluids measured were 353°C at E2 vent and 383°C at E9 (Table1). Both vent fields had a similar pH of ca. 3, but differed in their chemistry. Recent volcanic activity possibly led to very low chloride and high H_2_S concentrations at E9 and particularly the southern (E9S) part of the E9 vent field (James et al. 2014). Vent fluids in the northern part of the E9 vent field (E9N) were hotter and slightly lower in H_2_S than E9S (Table1). However, these differences were very small in comparison with the differences to the E2 vent field, which had roughly seawater chloride concentrations, lower H_2_S, and a lower Fe:Mn ratio, all characteristic of sub-surface cooling. Despite the differences in the end-member vent fluids, within the diffuse flow areas around the chimneys, where most *Kiwa* were situated, concentrations of various chemicals as well as physical properties were very similar between E2 and E9 (Table1).

**Table 1:** Location and properties of the sample sites (average of 2 measured values is shown, unless the values differed by more than 20%, in which case both values are shown)

	E2-Anemone field	E2-Crab city	E9 South-Marshland	E9 North-Black&White	Seawater^1^
Latitude	56°5.31 S	56°4.80 S	60°2.79 S	60°02.57 S	
Longitude	30°19.07 W	30°18.60 W	29°58.71 W	29°58.92 W	
Depth (m)	2597	2641	2402	2400	
Measurements in the diffuse flow near *Kiwa*					
pH	7.59	6.39	7.37/5.91	5.96	7.5–8.4
Temperature (°C)	3.5	20	5/20	11	∼0
H_2_S (mmol/L)	0	0.12	0/0.1	0.1	<0.000002
SO_4_ (mmol/L)	27.2	26.5	26.7	27.6	28
Cl (mmol/L)	531	541	525	539	546
Mg (mmol/kg)	52.7	49.9	51.5	52.0	52.7
Mn (*μ*mol/kg)	9.76	92.9	0.82/18.9	4.18	0.00036
Fe (*μ*mol/kg)	2.17	4.13	1.2/5.4	5.57	0.0005

	E2–Dog'sHead	E2-Sepia	E9 South – Ivory Tower	E9 North- Black&White
Measurements in the end-member fluid of the nearest vent chimney				
pH	3.02	3.05	3.08	3.42
Temperature (°C)	351	353	348	383
H_2_S (mmol/L)	6.7	7.1	11	9.5
Cl (mmol/L)	536	532	220	98.2
Mn (*μ*mol/kg)	2050	2060	508	199
Fe (*μ*mol/kg)	1280	1010	2520	800

1 Average values in seawater. Source: http://www.mbari.org/chemsensor/pteo.htm).

### Microscopic analysis of epibionts

The setae of the *Kiwa* sp. nov. ESR were densely covered in bacteria, with a mostly filamentous cell morphology (Fig.1C–E). There were no obvious differences in cell morphology or cell density of the epibionts of E2 and E9 *Kiwa* sp. nov. ESR specimen. However, it should be noted that at E2 several crabs were observed further away (within 100 m) from actively venting chimneys. These individuals had notably fewer epibionts. They were mostly females and the majority of them were carrying eggs (Marsh 2014). All sequencing and isotope analysis were carried out on specimen collected in close proximity to the vents.

### Identification of the epibionts by Sanger sequencing

**Figure 2:**
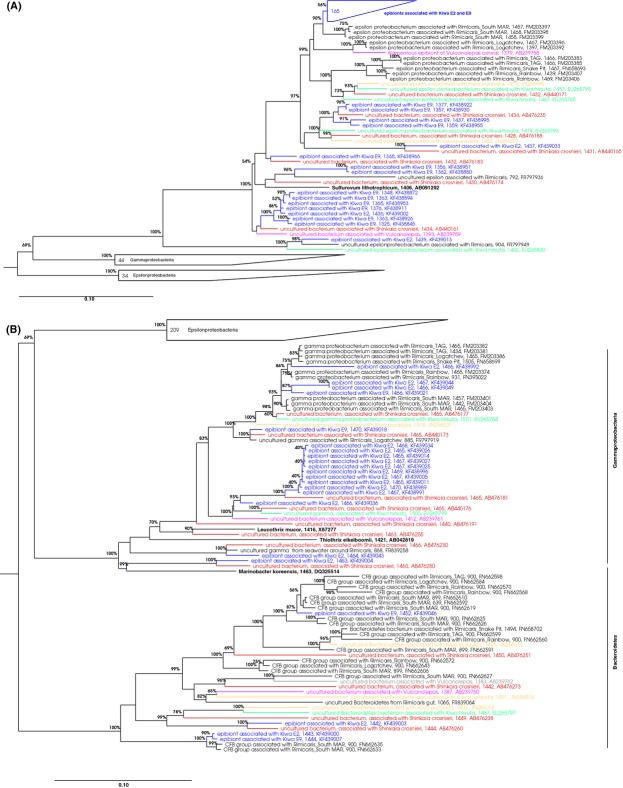
Neighbor joining tree of 16S rRNA sequences of *Kiwa* sp. nov. ESR (blue), *Kiwa hirsuta* (green), *Kiwa puravida* (yellow), *Shinkaia crosnieri* (red), *Rimicaris exoculata* (black), and *Vulcanolepas* sp. (pink). (A) *Epsilonproteobacteria*, (B) *Gammaproteobacteria* and *Bacteroidetes*. The tree was calculated within arb using neighbor joining with the Felsenstein correction using only sequences >1300 nt. Shorter sequences were then added by parsimony within arb. The length of each sequence is shown in the tree, followed by the Genbank accession number. Bootstrap values were calculated with the arb parsimony interactive tool. Only bootstrap values >50% are shown.

Sanger sequencing was used for a detailed phylogenetic classification of the dominant epibionts based on full length 16S rRNA sequences, while the 454 sequencing approach (see below) was used to compare the relative abundances of dominant phylogenetic groups and potential presence of rare taxa among epibionts from different *Kiwa* sp. nov. ESR specimen, sites, and vent fields. DNA was extracted from the epibionts of one E2 and one E9 *Kiwa* and clone libraries of the 16S rRNA gene were generated and sequenced. A total of 45 sequences from E2 and 165 sequences from E9 were analyzed (Table S1 and Fig.2). At E2, there was a roughly even distribution between *Gamma*- and *Epsilonproteobacteria*, as well as a few representatives from the *Bacteroidetes*. In contrast, at E9 the vast majority of sequences (97%) was assigned to the *Epsilonproteobacteria*. Most of these sequences clustered into three distinct phylotypes (i.e., with sequences within a phylotype showing a sequence identity of ≥97%). Two of the phylotypes were found at both E2 and E9, the third one was only found at E2 (Fig.2A).

Within the *Epsilonproteobacteria*, all sequences from E2 and E9 fell into the *Campylobacterales-Sulfurovum* cluster, with *Sulfurovum lithotrophicum* as the closest cultured relative. The majority of E2 and E9 sequences within the *Epsilonproteobacteria* (87%) clustered into one distinct phylotype, which has a moderate sequence identity with *S. lithotrophicum* of 93.3%. More closely related to this dominant phylotype, with a sequence identity of 98.6%, are several sequences from uncultured epibionts associated with *R. exoculata* derived from the South vent field in the Mid Atlantic Ridge (South MAR) (Petersen et al. 2010). The dominant *Kiwa* sp. nov. ESR epibiont and *R. exoculata* South MAR epibiont form a separate cluster from other epibionts of various species of vent fauna, such as those associated with the other two currently described yeti crabs *K. hirsuta* and *K. puravida,* the more distantly related crab *S. crosnieri,* the stalked barnacle *Vulcanolepas osheai,* as well as *R. exoculata* epibionts from other MAR vent fields (Fig.2A).

Within the *Gammaproteobacteria*, sequences were assigned to the *Thiotrichales-Thiothrix-Leucothrix* cluster, with 81.8–89.9% sequence identity with *Leucothrix mucor* as the closest cultured relative. The dominant phylotype of E2 epibiont sequences has epibionts of *S. crosnieri* (94.6% sequence identity) and *K. hirsuta* (94.9%) as the closest uncultured relatives. Most of the other E2 and E9 epibiont sequences are more closely related to *R. exoculata* epibionts, including ones from the South MAR location, than to epibionts of other vent species (Fig.2B).

Within the *Bacteroidetes*, three of the four retrieved *Kiwa* sp. nov. ESR epibiont sequences have *R. exoculata* epibionts as the closest relative, with two of the sequences showing high similarity (98.3%) to South MAR epibionts (Fig.2B).

### Comparison of E2 and E9 *Kiwa* sp. nov. ESR epibionts based on 454 pyrosequencing data

**Figure 3:**
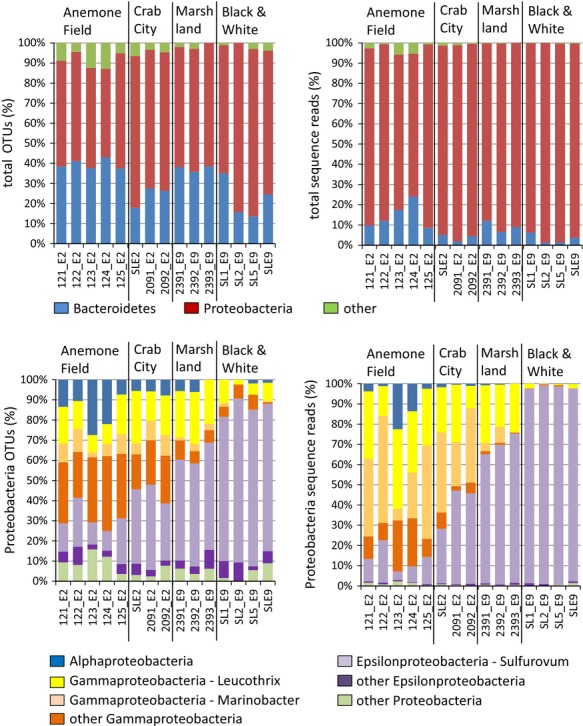
Phylogenetic analysis of 454 sequencing data of epibionts from 15 *Kiwa* sp. nov. ESR individuals. OTUs are defined at a cutoff of 0.03. ESR, East Scotia Ridge; OTUs, operational taxonomic units.

16S rDNA amplicon sequencing was carried out with epibionts from 15 individuals (eight from E2 and seven from E9). This generated a data set of 34,183 sequences after trimming and quality control. *Kiwa* sp. nov. ESR were collected at two different sites at E2 (Anemone field and Crab city) and two sites at E9 (E9S-Marshland and E9N-Black&White) (Table2 and Fig.3).

**Table 2:** Details of the *Kiwa* sp. nov. ESR specimen, which were used for 454 analysis of the epibionts

Sample	OTUs	Sequences	Coverage	Gender	Carapace length (cm)	Sample site	Shannon
121_E2	283	2513	0.938	Male	9	E2 – Anemone field	3.196
122_E2	226	2522	0.956	Male	7.5	E2 – Anemone field	2.891
123_E2	329	2020	0.907	Female	5.5	E2 – Anemone field	4.287
124_E2	372	2317	0.916	Female	5.5	E2 – Anemone field	4.392
125_E2	285	2735	0.944	Female	6.5	E2 – Anemone field	2.932
2091_E2	168	2412	0.960	Female	5.5	E2 – crab city	2.387
2092_E2	178	1987	0.951	Female	4.5	E2 – crab city	2.715
SLE2	281	4019	0.970	Female	7	E2 – crab city	2.825
2391_E9	210	2894	0.963	Juvenile	3	E9 South – Marshland	2.958
2392_E9	134	1800	0.961	Juvenile	3	E9 South – Marshland	2.619
2393_E9	52	549	0.949	Juvenile	3	E9 South – Marshland	2.331
SL1_E9	94	1264	0.960	Male	5.5	E9 North – B&W	1.717
SL2_E9	51	769	0.964	Male	6	E9 North – B&W	1.269
SL5_E9	66	1161	0.974	Juvenile	3	E9 North – B&W	1.360
SLE9	187	5221	0.987	Juvenile	3	E9 North – B&W	2.177
Total		34,183					

ESR, East Scotia Ridge; OTUs, operational taxonomic units.

Overall Shannon diversity was higher in the E2 samples than the E9 samples. As expected based on the Sanger sequencing results, *Proteobacteria* and *Bacteroidetes* were the dominant phyla at all sites, with *Proteobacteria* accounting for 70.6–98.7% of all sequence reads and 44.1–84.3% of all operational taxonomic units (OTUs) (at 0.03 cutoff) and *Bacteroidetes* accounting for 1.3–24.1% of sequence reads and 13.6–43.0% of OTUs (Fig.3 and Table S2). The striking contrast between the *Epsilonproteobacteria*-dominated E9 epibionts and the *Epsilon*-/*Gammaproteobacteria* mix of E2 epibionts found with Sanger sequencing was supported with the increased replication that was undertaken with the 454 data for a larger number of samples derived from four different sampling sites. There was a high consistency of the phylogenetic composition of samples from the same site. Across the samples from the E9N Black & White site 94.4% (standard deviation 2.8) of all sequence reads were identified as *Epsilonproteobacteria*. At the E9S sampling site Marshland the proportion of *Epsilonproteobacteria* was lower (63.1%, SD 4.8) and *Gammaproteobacteria* were more abundant (26.7%, SD 3.2). At E2 the ratio was shifted more toward *Gammaproteobacteria*, with 55.7% (SD 7.0) and 64.5% (SD 9.0) at the E2 sites Crab City and Anemone Field compared to just 38.1% (SD 8.7) and 10.3% (SD 5.4) of *Epsilonproteobacteria* at these sites.

Notable is the appearance of up to 17.3% *Alphaproteobacteria* in the samples from E2 – Anemone Field. Within the *Epsilonproteobacteria*, sequences were almost exclusively assigned to the *Campylobacterales-Sulfurovum* cluster, while there was a split between *Thiotrichales-Thiothrix-Leucothrix* and *Alteromonadales-Marinobacter* within the *Gammaproteobacteria*. No *Marinobacter* sequences were found with the Sanger sequencing approach, although this may be due to the limited number of clones analyzed. In contrast to the *Epsilon*- and *Gammaproteobacteria*, where the majority of epibionts clustered into one group (represented by several phylotypes), the *Bacteriodetes* epibionts were widespread across several orders and multiple families with only distant similarities (<85%) to cultured species, but high similarities (>95%) to epibionts of other vent fauna, particularly *R. exoculata*. The most common *Bacteroidetes* families found among the *Kiwa* ESR epibionts were *Saprospiraceae*, *Chitinophagaceae,* and *Flavobacteriaceae*.

**Figure 4:**
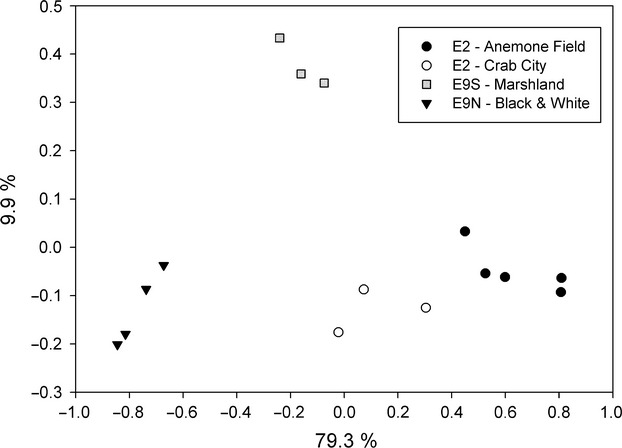
PCoA of *Kiwa* sp. nov. East Scotia Ridge (ESR) epibionts of 15 specimen at four E2 and E9 sampling sites based on 454 data.

Principal coordinates analysis (PCoA) (Fig.4) confirmed the split between E2 and E9 epibiont communities and also showed a strong clustering of epibionts from *Kiwa* sp. nov. ESR collected at the same site. The epibiont community of samples from each site was significantly different (*P* < 0.001) from the epibiont communities of samples from the other sites (*P*-test, Martin 2002).

### Isotopic niches of *Kiwa* sp. nov. ESR at E2 and E9

**Figure 5:**
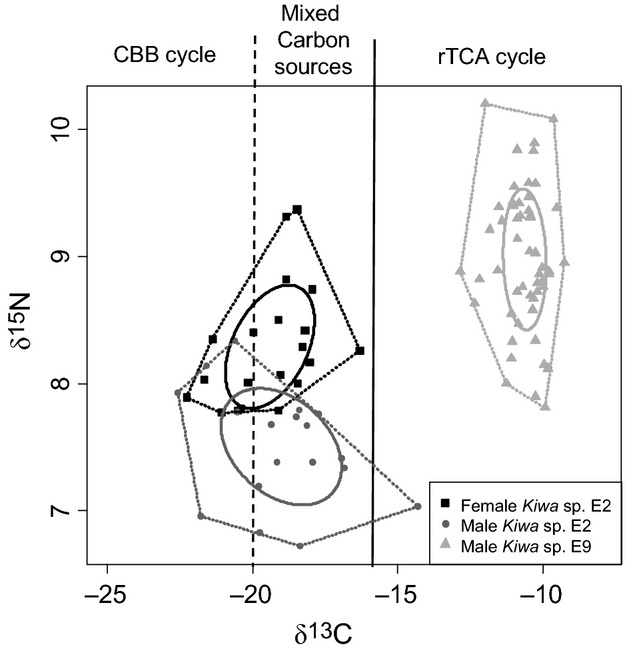
Isotopic niche of *Kiwa* sp. nov. East Scotia Ridge (ESR) *δ*^13^C and *δ*^15^N values for female (squares) and male (circles) *Kiwa* sp. nov. ESR from E2 and male (triangle) *Kiwa* sp. nov. ESR from E9 with their respective standard ellipse area (solid line) with a boundary plotted (dotted line) around the extreme points to aid visualization. The dashed and solid vertical lines represent the range of *δ*^13^C values for different carbon fixation pathways: <−20‰ for the Calvin-Benson-Bassham (CBB) cycle, >−16‰ for the reductive tricarboxylic acid (rTCA) cycle and −20‰ to −16‰ for mixing of the two carbon sources.

The isotopic niche area (‰^2^), as defined by a sample size SEAc, overlapped by only 18% between female and male *Kiwa* sp. nov. ESR at E2 (Fig.5). Bayesian inference showed an 80% probability that males had a greater isotopic niche area than females at E2. Neither E2 male nor female isotopic niche area overlapped with the niche area of males collected from E9 (Fig.5). Bayesian inference indicated that there was *a* > 98% probability that E2 males and female isotopic niche area were greater than E9 male *Kiwa* sp. nov. ESR.

### Trends related to the size of the host

Within the E2 vent field, the sampled female *Kiwa* sp. nov. ESR were significantly smaller (CL) than the males (*t*-test, *n* = 37, *P* < 0.01). The population at E9 was dominated by males and juveniles of undetermined sex and we were not able to collect females for stable isotope or epibiont sequence analysis. A comparison of male *Kiwa* sp. nov. ESR at E2 and E9 showed that the E2 individuals were significantly larger than those at E9 (*t*-test, *n* = 69, *P* < 0.01). At the same time, the Shannon diversity index (Table2) of the epibionts was higher at E2 than at E9 (*t*-test, *n* = 15 [including male, female and juvenile hosts], *P* < 0.01).

**Figure 6:**
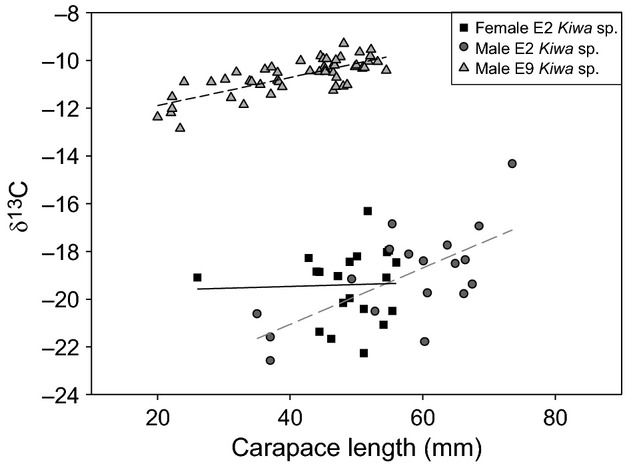
Plots of *δ*^13^C against carapace length (mm) for *Kiwa* sp. nov. East Scotia Ridge (ESR) for samples collected from E2 female (squares and solid line), E2 male (circles and long dash line), and E9 male (triangles and short dash line) *Kiwa*. In male *Kiwa* sp. nov. ESR there was an increase in *δ*^13^C with carapace length (mm) (CL) in E2 (*δ*^13^C = −25.78 + 0.118 CL; *F*_1,16_ = 12.93, *r*^*2*^_adj_ = 0.41, *P* < 0.01) and E9 (*δ*^13^C = −13.06 + 0.059 CL; *F*_1,50_ = 67.81, *r*^*2*^_adj_ = 0.58, *P* < 0.001). However, female *Kiwa* sp. nov. ESR did not increase in *δ*^13^C with CL (*δ*^13^C = −21.52 + 0.118 CL; *F*_1,17_ = 0.2428, *r*^*2*^_adj_ = −0.04, *P* = 0.69).

*δ*^13^C values increased with CL in male E9 *Kiwa* and male E2 *Kiwa*, but not in female E2 *Kiwa* (Fig.6).

## Discussion

### Phylogenetic diversity of the epibiont communities

The vast majority of the *Epsilonproteobacteria* among the *Kiwa* sp. nov. ESR epibionts fell into the *Sulfurovum* cluster. Members of the genus *Sulfurovum* are considered to be important primary producers in chemosynthetic systems (Campbell et al. 2006) and have been found living on various species of vent fauna close to active venting (Goffredi et al. 2008; Goffredi 2010; Petersen et al. 2010; Tsuchida et al. 2011). A recent metatranscriptomic analysis of a *Sulfurovum* dominated biofilm at Loki's Castle vent field on the MAR (Dahle et al. 2013) showed expression of genes involved in respiration with sulfur species, hydrogen, formate, nitrate, and oxygen. Particularly common were transcripts for cbb3-type cytochrome c oxidase, which supports microaerobic respiration, as well as transcripts of the sox gene cluster, responsible for oxidation of sulfur compounds. The presence of transcripts of the ATP citrate lyase and other key enzymes suggested C-fixation via the reverse tricarboxylic acid (rTCA) cycle.

Within the *Gammaproteobacteria*, the genera *Leucothrix* and *Marinobacter* were identified as the closest relatives (albeit with only moderate sequence identity) for a large proportion of the epibiont sequences at E2. *Marinobacter* is abundant in hydrothermal vent environments, particularly within metal sulfide deposits (Rogers et al. 2003; Kaye et al. 2011) and in water samples among mussels in the Logatchev vent field at MAR (Perner et al. 2013). To our knowledge, *Marinobacter* has not previously been found as a common component of epibiont communities. Physiological studies on *Marinobacter* isolates from the Juan de Fuca Ridge identified them as iron oxidizing, microaerophilic and obligate chemolithoautotrophs and suggested a role in the weathering of metal sulfide deposits (Edwards et al. 2003).

*Leucothrix* is also commonly found at hydrothermal vents, including the epibiont communities of *K. hirsuta*, *S. crosnieri,* and *R. exoculata*. The closest cultured relative, *L. mucor,* has been shown to be a chemolithoheterotroph, oxidizing thiosulfate (Grabovich et al. 1999). However, a study on *R. exoculata* showed evidence for the cbbM gene in the *Leucothrix*-containing epibiont community in the mouthparts of the shrimp (Hügler et al. 2011). The cbbM gene codes for RubisCO form II and is thus indicative for the capability of autotrophic growth via the CBB cycle. This was confirmed in a recent study of the metagenome of *R. exoculata* epibionts, which showed the presence of a complete set of genes for the CBB cycle in the *Leucothrix*-like epibionts (Jan et al. 2014). Therefore, a mixotrophic life-style of *Leucothrix*-like epibionts in vent systems has been suggested (Hügler et al. 2011).

Sequence identity of the *Kiwa* sp. nov. ESR epibionts with both *Sulfurovum* and *Leucothrix* is only moderate and deductions on the metabolic capabilities of the epibionts therefore uncertain. The closest relatives among uncultured bacteria for the dominant *Epsilonproteobacteria* phylotype as well as *Gammaproteobacteria* and *Bacteroidetes* sequences of the *Kiwa* sp. nov. ESR epibionts are not epibionts of other *Kiwa* species, but rather *R. exoculata* epibionts from the South MAR vent field. Petersen et al. (2010) analyzed *R. exoculata* epibionts from several vent fields along MAR and found a correlation between genetic distance of the epibionts and geographic distance. Interestingly, in that study the South MAR *R. exoculata* epibionts were shown to form a separate group from *R. exoculata* epibionts from other MAR vent fields. The geographic distance between the South MAR vent field and E9-ESR is very similar to the distance between South MAR and Rainbow (the northernmost MAR vent field analyzed by Petersen et al.), ca. 8500 km along the ridges. This points to a genetic link between the epibionts at ESR and South MAR with potentially several currently unknown vent sites along the ridges as stepping stones. The geographic distance along the edges of tectonic plates with known or unknown hydrothermal activity is greater between *Kiwa* sp. nov. ESR and either *K. hirsuta*, *K. puravida* or *S. crosnieri* than between *Kiwa* sp. nov. ESR and *R. exoculata* South MAR. This could explain why the *Kiwa* sp. nov. ESR epibionts were shown to be genetically more similar to *R. exoculata* South MAR epibionts than epibionts of any of the other vent species analyzed here.

### Epibionts as food source for *Kiwa* sp. nov. ESR

The role of epibionts as a food source has been discussed for several vent and seep species, such as *S. crosnieri* (Watsuji et al. 2010; Tsuchida et al. 2011), *K. puravida* (Thurber et al. 2011), *K. hirsuta* (Goffredi et al. 2008) and *R. exoculata* (Polz and Cavanaugh 1995; Petersen et al. 2010; Ponsard et al. 2013). Evidence for this is based on isotopic signatures of epibionts and hosts (Van Dover 2002; Suzuki et al. 2005), uptake and transfer of isotope labeled inorganic carbon (Ponsard et al. 2013), fatty acid analysis (Suzuki et al. 2005; Thurber et al. 2011) as well as behavioral observations like the “dancing for food” (i.e., waving the epibiont-covered arms in nutrient rich seep fluid) described by Thurber et al. (2011) and combing epibionts toward the mouth (Thurber et al. 2011; Tsuchida et al. 2011).

Assuming that epibionts serve as a food source, differences in the epibiont community composition have implications for the *δ*^13^C values of associated bulk organic carbon of both epibionts and host. *Gamma*- and *Epsilonproteobacteria* fix carbon via different metabolic pathways, the CBB and rTCA cycle, respectively, resulting in isotopically distinct *δ*^13^C values (Hügler et al. 2011). Organic carbon produced via the rTCA cycle at hydrothermal vents tends to have values >−16‰, while values between −20‰ and −30‰ are believed indicative of the CBB cycle (Hügler et al. 2011).

We found clear differences in isotopic niches of *Kiwa* between E2 and E9 (Fig.5), which indicated that there were differences in the food source that was assimilated. At the same time, phylogenetic sequence analysis of the epibionts also showed striking differences between E2 and E9 (Fig.3). At E9, the *δ*^13^C values of the *Epsilonproteobacteria,* which dominated the epibiont community, (−9.9‰ SD 0.3, *n* = 5) indicate carbon fixation via the rTCA cycle (Reid et al. 2013). Furthermore, the *δ*^13^C values of E9 *Kiwa* (−10.6‰ SD 0.7, *n* = 52) also lie within the range reported for rTCA cycle (Reid et al. 2013) and therefore suggest a direct trophic link between the epibionts and *Kiwa* sp. nov. ESR.

At E2, the mean *δ*^13^C values for epibionts (−18.9‰ SD 5.3, *n* = 5) and host (−19.2‰ SD 1.7, *n* = 38) suggest a direct trophic link between epibionts and host, but they are slightly higher than the reported values for carbon fixation exclusively via CBB. It is likely that the *δ*^13^C values are the result of assimilation of a mix of CBB and rTCA fixed carbon. Following the hypothesis that the epibionts serve as a food source, both the lower mean and the broader range of *δ*^13^C values in E2 *Kiwa* compared to E9 *Kiwa* (Figs.5, 6) can be seen as an indication of a more diverse food source that reflects the spatial differences in the epibiont community associated with the ventral setae. Epibiont sequence data, showing a mix of *Gamma*- and *Epsilonproteobaceria* at E2 *Kiwa,* confirmed this. Taken together, the co-varying isotope values and epibiont diversity provide strong support for the food source hypothesis.

There was no evidence for *Kiwa* sp. nov. ESR preying on other vent animals. Grazing on bacterial mats around the vents could be another potential food source for the crabs. Unfortunately we do not have any sequence data for bacterial mats at ESR. Isotope analysis of rock scrapings at E9 and particulate suspended material at E2 (Reid et al. 2013) does not suggest a trophic link between bacteria from these substrates and *Kiwa* sp. nov. ESR*,* although it should be noted that the number of samples analyzed was too small to allow conclusive statistical analysis.

### Differences in epibiont community composition within and between the ESR vent fields

Regional variations in the community composition of the epibionts are likely the result of a combination of factors. We propose that the main driving forces, in decreasing order of importance, are:

*1. Geology and geochemistry of the vents*. Geologically, E2 is characterized by more mature chimney structures and there are indications of sub-surface cooling, which can influence the chemical composition of the vent fluids (Seyfried and Ding 1993). E9, on the other hand, appears to be hydrothermally more active, possibly following a recent volcanic eruption, and is characterized by nascent chimneys and extremely low Cl- concentrations.

Differences in the chemical environmental conditions at E2 and E9 could account for some of the phylogenetic differences in the epibiont communities. It should be noted that the exact environmental parameters within the diffuse flow at the point where the *Kiwa* sp. nov. ESR were sampled were difficult to determine, as the location of the temperature probe, the Ocean Test Equipment (OTE) bottles for taking water samples and the robotic arms for collecting *Kiwa* sp. nov. ESR are located on different positions on the ROV and variation can occur at spatial scales of <1 m. The values given in Table1 for the diffuse flow therefore serve as a guideline. More conclusive are the measurements of the end-member fluids, which show distinct differences in the overall chemistry between E2 and E9. These fluids are diluted with seawater in sub-surface mixing before emission into the deep ocean in the diffuse flow areas.

The E9 vent field, and particularly E9N, is characterized by higher H_2_S and lower Cl- concentrations compared to E2 (Table1 and Rogers et al. 2012). This may benefit the sulfur oxidizer *Sulfurovum*. E2, on the other hand, has overall lower H_2_S concentrations, higher concentrations of Fe and especially high concentrations of Mn (Table1 and James et al. 2014). Members of the genus *Marinobacter* have been shown to be Fe oxidizers (Edwards et al. 2003) and at least one species, *Marinobacter manganoxydans*, is known to oxidize Mn and is able to tolerate extreme Mn concentrations (Wang et al. 2012).

*2. Size (age) and moulting stage of the host. Kiwa* at E2 were on average larger and had a more diverse epibiont community than *Kiwa* at E9, where a large proportion of the population consisted of juveniles. Using size as a proxy for age, this could be an indication that the epibiont community composition changes with age. This has been described in *R. exoculata* (Guri et al. 2012) and more recently in *K. puravida* (Goffredi et al. 2014). In both cases, a shift from a *Gammaproteobacteria* dominated to an *Epsilonproteobacteria*-dominated epibiont community was observed from egg to adult. We did not analyze any eggs or larvae of *Kiwa* sp. nov. ESR, so may only see later stages of a possible shift. However, the clear increase of *δ*^13^C values with size, within both E2 and E9 (Fig.6), indicates a change in the food source of *Kiwa* sp. nov. ESR and therefore putatively a change of their epibionts. As *δ*^13^C increases with size, this would suggest that more carbon is being assimilated that was fixed via the rTCA cycle. Length-based trends in *δ*^13^C are also found in other caridean vent shrimps (Polz et al. 1998; Van Dover 2002). For example, *R. exoculata* increases in *δ*^13^C by 6–7‰ with increasing length as it changes from a photosynthetic diet during larval and juvenile stages to a chemosynthetic diet as an adult (Polz et al. 1998; Vereshchaka et al. 2000; Van Dover 2002). The *δ*^13^C-length trends in *R. exoculata* are in contrast to the gradual increase in *δ*^13^C with size observed in *Kiwa* sp. nov. ESR sp. Even though *Kiwa* sp. nov. ESR *δ*^13^C-length relationships varied with site, the trends still suggest they were not driven by a dilution of photosynthetic primary production over the size range sampled but are reflective of a change in the utilization of carbon within the hydrothermal vent. Despite the 454 sequencing sample size not being large enough to draw reliable conclusions regarding a correlation between microbial diversity and size, the much greater sample size for stable isotope analysis (*n* = 90) would suggest that changes in epibiont communities with size or gender is a feasible hypothesis.

The *Kiwa* moulting cycle may also have an effect on the epibiont community composition. After each moulting, epibiotic bacteria have to recolonize their host. In *Rimicaris,* this recolonization involves a succession of different bacteria (Corbari et al. 2007; Guri et al. 2012). At E2, *Kiwa* generally had a visibly “dirtier” carapace than *Kiwa* at E9 and showed some necrosis of the setae, which is indicative of a later stage of the moulting cycle (Sven Thatje, pers. comm. 2013). This could account for the stark contrast between the *Epsilonproteobacteria*-dominated E9 epibionts and the *Gamma-/Epsilonproteobacteria* mix at E2.

*3. Differential behavior of male and female Kiwa* sp. nov. ESR. At E2, the *δ*^13^C values of *Kiwa* sp. nov. ESR showed a broader range than at E9 as well as a distinct split between males and females (Figs.5, 6).

This may be due to changes in behavior and location throughout the life cycle of the crabs (Marsh 2014). While juveniles (particularly at E9) were generally found sitting in diffuse flow regions at the bottom of actively venting chimneys, larger, and especially large male *Kiwa* sp. nov. ESR were often observed climbing toward the top of a chimney (Marsh et al. 2012), where they are likely to be exposed to different chemical and physical environmental conditions. At E2, females were frequently observed further away from active venting and outside areas of diffuse flow, resulting again in different environmental conditions. A large number of these females were carrying eggs (Sven Thatje, pers. comm 2010). At the same time, these individuals had noticeably fewer epibionts (K. Zwirglmaier, pers. obs. 2010), underlining the dependence of the epibionts on the environmental conditions in close proximity to the vents. No sequence data are available for these off-site individuals and at this point it is unknown, whether adult *Kiwa* sp. nov. ESR regularly move from these extreme locations (off-site or on top of chimney) to the more temperate conditions within the diffuse flow.

## Summary and Conclusion

This study presents the first description of the epibiont community of a new species of *Kiwa* from the first hydrothermal vent system discovered in the Antarctic. High throughput sequencing analysis of the epibionts of 15 individuals revealed strong regional differences between the two studied vent fields, which were also reflected in *δ*^13^C values. The combination of the sequencing results with *δ*^13^C values of both the hosts and the epibionts provided insights into the putative carbon fixation pathways of the epibionts and the symbiotic relationship with their hosts and supports the hypothesis that the epibionts serve as a food source. Near full-length 16S rRNA Sanger sequencing helped to clarify the phylogenetic affiliation of the major groups identified with the shorter 454 sequences and showed that most phylotypes of the *Kiwa* sp. nov. ESR epibionts are closely related to *R. exoculata* epibionts from the South MAR vent field. The major *Epsilonproteobacteria* epibionts of *Kiwa* sp. nov. ESR form a subgroup together with the *R. exoculata* South MAR epibionts that is separate from epibiont communities of other vent fauna.

Future work on the metagenome and/or metatranscriptome in combination with further (geo)chemical analysis of the vent fields is required to further describe the epibiotic relationship of the microorganisms and the host and define their role in the nutrient cycles of this vent system.

## Supporting Information

**Data S1.** Supplementary information. **Table S1.** Comparison of the phylogenetic association of near full-length (>1300 nt) 16S rRNA sequences of epibionts of *Kiwa* sp. nov ESR at E2 and E9. **Table S2.** 454 sequence data. (A) % sequence reads, (B) % OUT at 0.03 cutoff. **Table S3.** Details of *Kiwa* sp. nov ESR specimen collected for stable isotope analysis.
