# Supplementary material for: Linking regional variation of epibiotic bacterial diversity and trophic ecology in a new species of Kiwaidae (Decapoda, Anomura) from East Scotia Ridge (Antarctica) hydrothermal vents
Source: Microbiologyopen. 2014 Dec 16;4(1):136–50. doi: 10.1002/mbo3.227 (PMC4335981; doi:10.1002/mbo3.227)
Supplement: Supplementary file 1 [file mbo30004-0136-sd1.doc]

**Linking regional variation of epibiotic bacterial diversity and trophic ecology in a new species of Kiwaidae (Decapoda, Anomura) from East Scotia Ridge (Antarctica) hydrothermal vents**

K. Zwirglmaier , W. D. K. Reid , J. Heywood , C. J. Sweeting , B. D. Wigham , N. V. C. Polunin

J. A. Hawkes , D. P. Connelly , D. Pearce , K. Linse

**Supplementary information**

**Material and Methods**

*DNA extraction*

Setae covered with epibiotic bacteria were cut off from the ventral side of the *Kiwa* sp. nov. ESR with sterilised scissors and suspended in 750 µl TE buffer (10 mM Tris, 1 mM EDTA, pH 8.0) in a 2ml tube. After adding 30 µl of Proteinase K (800U/µl), 30µl lysozyme (50 mg/ml) and 75 µl of a 1% SDS solution the sample was incubated for 2 h at 37°C. 750µl of phenol/chloroform/isoamylalcohol (25/24/1) were then added and the tube was shaken vigorously, and then centrifuged for 2 min at 13000 x g. The upper (aqueous) phase was transferred to a fresh 2ml tube, 1 volume of chloroform was added, and the tube was shaken vigorously and then centrifuged again for 2 min at 13000 x g. The upper phase was transferred to a fresh 2 ml tube and the DNA was precipitated by adding 1 vol. of isopropanol and 0.1 vol. of sodium acetate (3 M, pH 5.5). To pellet the DNA, the tube was centrifuged 15 min at 13000 x g at 4°C. The pellet was washed with 70% ethanol and finally resuspended in TE buffer.

*454 sequence analyis*

A combination of arb and mothur v. 18.0 was used to analyse the 454 data.

Quality control of the sequences was carried out within mothur. Sequences were trimmed based on the qfile, with a qwindowaverage of ≥30 and a windowsize of 50. Sequences with any ambiguities or homopolymers longer than 10 nt or a length <100nt were discarded. Chimeric sequences were detected and discarded with chimera slayer within mothur. The trimmed set of sequences was aligned in arb using SINA and a distance matrix was created within arb using the Jukes-Cantor correction. Sequences were then clustered with mothur using the furthest neighbour method. OTUs were defined at the level of 0.03. Sequences were classified with mothur to assign a taxonomy using a reference database containing 16073 16S rRNA sequences, which is based on the living tree project database LTPv104 , containing 8545 sequences, plus 7528 hydrothermal vent related sequences downloaded from Silva .

Shannon diversity index and OTU coverage of each sample (Table 2) were also calculated within mothur. Principal coordinate analysis (PCoA)(fig. 4) was done with FastUnifrac . The phylogenetic tree used for PCoA was generated in arb by adding the 454 data to the LTPv104 tree by parsimony and then deleting the LTP sequences from the tree.

*Stable isotope data analysis*

The dispersion of δ^13^C and δ^15^N values in *xy*-space were parameterised by calculating the total area (TA) and standard ellipse area (SEA) using the SIAR package implemented in the R statistical package version 2.13.2. TA encompasses all the data points within its area calculation by delineating a boundary around the extreme data points and thus can only become greater with increasing sample size . The TA is used, here, to demark the extreme points in order to aid in the visualisation of these data. The SEA is calculated from the variance and covariance of a normally distributed bivariate data set. The SEA contains approximately 40% of these data and represents the isotopic values of the core of the population or sample. SEA is robust to varying sample sizes and is therefore preferred when comparing isotopic niches amongst groups .

The standard ellipses reported here are corrected for small sample sizes (SEAc) as described by . The overlap between SEAc was calculated, which provides a quantitative measure of isotopic niche similarity . The SEA can also be compared through a Bayesian inference. A matrix of Bayesian standard ellipse areas (SEA_B_) was created based on 10000 posterior iterations and the proportion of SEA_B_ that differ between two groups calculated. The results can be interpreted as the probability that the posterior distribution of the model parameters given the prior data that one group occupies a larger isotopic niche than the other). The Bayesian method allows a direct probabilistic interpretation of the differences in SEA_B_ by pair-wise comparisons .

Suppl Table 1: Comparison of the phylogenetic association of near full-length (>1300nt) 16S rRNA sequences of epibionts of *K.* sp. nov ESR at E2 and E9

|  | *Kiwa* E2 epibionts | *Kiwa* E9 epibionts |
| --- | --- | --- |
| Number of sequences | 45 | 165 |
| *Gammaproteobacteria* | 17 | 3 |
| of which *Leucothrix* | 15 | 3 |
| *Epsilonproteobacteria* | 22 | 160 |
| of which *Sulfurovum* | 22 | 158 |
| Other | 6 | 2 |
| % *Gammaproteobacteria* | 38 | 2 |
| % *Epsilonproteobacteria* | 49 | 97 |
| % other | 13 | 1 |

| % seq reads |  | |  |  |  |  |  |  |  |  |  |  |  |  |  |
| --- | --- | --- | --- | --- | --- | --- | --- | --- | --- | --- | --- | --- | --- | --- | --- |
|  | Anemone Field | | | | | Crab City | | | Marshland | | | Black & White | | | |
|  | **121_E2** | **122_E2** | **123_E2** | **124_E2** | **125_E2** | **SLE2** | **2091_E2** | **2092_E2** | **2391_E9** | **2392_E9** | **2393_E9** | **SL1_E9** | **SL2_E9** | **SL5_E9** | **SLE9** |
| **total** | 100 | 100 | 100 | 100 | 100 | 100 | 100 | 100 | 100 | 100 | 100 | 100 | 100 | 100 | 100 |
| **Bacteroidetes** | 9.8 | 12.1 | 17.5 | 24.1 | 8.6 | 5.1 | 1.9 | 4.5 | 12.1 | 6.7 | 8.9 | 6.3 | 1.3 | 1.4 | 3.8 |
| **Proteobacteria** | 87.5 | 87.3 | 76.7 | 70.6 | 90.6 | 93.7 | 97.1 | 95.0 | 87.8 | 93.0 | 91.1 | 93.7 | 98.7 | 98.4 | 95.9 |
| **Alphaproteobacteria** | 3.3 | 1.1 | 17.3 | 9.6 | 2.3 | 1.6 | 0.5 | 1.0 | 0.7 | 0.4 | 0.0 | 0.0 | 0.0 | 0.1 | 0.1 |
| **Gammaproteobacteria** | 72.5 | 66.5 | 54.0 | 54.1 | 75.4 | 65.6 | 50.9 | 50.5 | 29.9 | 27.7 | 22.4 | 2.4 | 0.7 | 1.4 | 2.2 |
| **Gammaproteobacteria - Marinobacter** | 33.6 | 46.3 | 4.4 | 15.9 | 41.9 | 36.9 | 21.1 | 35.0 | 3.6 | 7.3 | 0.2 | 0.1 | 0.0 | 0.0 | 0.0 |
| **Gammaproteobacteria - Leucothrix** | 29.1 | 12.7 | 30.1 | 21.5 | 25.3 | 21.1 | 27.6 | 10.5 | 25.0 | 19.3 | 21.9 | 2.1 | 0.3 | 0.6 | 2.1 |
| **other Gammaproteobacteria** | 9.7 | 7.4 | 19.4 | 16.8 | 8.1 | 7.6 | 2.2 | 5.1 | 1.3 | 1.1 | 0.4 | 0.2 | 0.4 | 0.7 | 0.0 |
| **Epsilonproteobacteria** | 10.3 | 19.1 | 3.7 | 5.7 | 12.6 | 25.8 | 45.2 | 43.2 | 56.6 | 64.5 | 68.1 | 91.2 | 98.0 | 96.1 | 92.2 |
| **Epsilonproteobacteria - Sulfurovum** | 9.7 | 18.3 | 3.2 | 5.5 | 12.1 | 25.3 | 44.8 | 42.4 | 56.0 | 64.1 | 67.2 | 90.0 | 97.1 | 96.0 | 91.4 |
| **other Epsilonproteobacteria** | 0.6 | 0.8 | 0.5 | 0.3 | 0.5 | 0.5 | 0.5 | 0.8 | 0.6 | 0.4 | 0.9 | 1.2 | 0.9 | 0.2 | 0.8 |
| **other Proteobacteria** | 1.4 | 0.7 | 1.8 | 1.1 | 0.4 | 0.7 | 0.5 | 0.3 | 0.6 | 0.3 | 0.5 | 0.1 | 0.0 | 0.8 | 1.5 |
| **other Bacteria** | 2.7 | 0.6 | 5.8 | 5.4 | 0.8 | 1.2 | 1.0 | 0.5 | 0.1 | 0.3 | 0.0 | 0.1 | 0.0 | 0.3 | 0.3 |
|  | Anemone Field | |  |  |  | Crab City |  |  | Marshland | |  | Black & White | |  |  |
| **average Epsilonproteobacteria (s.d.)** | 10.3 | (5.4) |  |  |  | 38.1 | (8.7) |  | 63.1 | (4.8) |  | 94.4 | (2.8) |  |  |
| **average Gammaproteobacteria (s.d.)** | 64.5 | (9.0) |  |  |  | 55.7 | (7.0) |  | 26.7 | (3.2) |  | 1.6 | (0.7) |  |  |

Suppl. Table 2. 454 sequence data. 2a) % sequence reads, 2b) % OUT at 0.03 cutoff

Suppl table 2b: % OTUs at 0.03% cutoff

| % OTUs | Anemone Field | | | | | Crab City | | | Marshland | | | Black & White | | | |
| --- | --- | --- | --- | --- | --- | --- | --- | --- | --- | --- | --- | --- | --- | --- | --- |
|  | **121_E2** | **122_E2** | **123_E2** | **124_E2** | **125_E2** | **SLE2** | **2091_E2** | **2092_E2** | **2391_E9** | **2392_E9** | **2393_E9** | **SL1_E9** | **SL2_E9** | **SL5_E9** | **SLE9** |
| **total** | 100 | 100 | 100 | 100 | 100 | 100 | 100 | 100 | 100 | 100 | 100 | 100 | 100 | 100 | 100 |
| **Bacteroidetes** | 38.5 | 41.2 | 37.7 | 43.0 | 37.5 | 17.9 | 27.5 | 26.3 | 38.1 | 35.8 | 38.5 | 35.1 | 15.7 | 13.6 | 24.6 |
| **Proteobacteria** | 52.7 | 54.4 | 49.8 | 44.1 | 57.2 | 75.6 | 69.1 | 69.0 | 60.0 | 61.2 | 61.5 | 63.8 | 84.3 | 83.3 | 71.7 |
| **Alphaproteobacteria** | 7.1 | 5.8 | 13.7 | 9.7 | 4.2 | 4.2 | 3.9 | 5.3 | 3.3 | 3.7 | 0.0 | 0.0 | 0.0 | 1.5 | 1.1 |
| **Gammaproteobacteria** | 30.4 | 26.1 | 21.6 | 23.4 | 35.1 | 36.9 | 32.0 | 37.0 | 20.5 | 21.6 | 19.2 | 11.7 | 7.8 | 12.1 | 7.5 |
| **Gammaproteobacteria - Marinobacter** | 4.9 | 6.2 | 1.2 | 2.7 | 5.6 | 4.2 | 6.7 | 7.1 | 1.0 | 2.2 | 1.9 | 1.1 | 0.0 | 0.0 | 0.5 |
| **Gammaproteobacteria - Leucothrix** | 9.5 | 7.5 | 4.3 | 4.3 | 11.2 | 19.6 | 10.1 | 13.5 | 13.8 | 15.7 | 13.5 | 7.4 | 2.0 | 4.5 | 6.4 |
| **other Gammaproteobacteria** | 15.9 | 12.4 | 16.1 | 16.4 | 18.2 | 13.1 | 15.2 | 16.4 | 5.7 | 3.7 | 3.8 | 3.2 | 5.9 | 6.1 | 0.5 |
| **Epsilonproteobacteria** | 10.2 | 18.1 | 6.7 | 5.6 | 15.8 | 32.1 | 31.5 | 21.4 | 32.4 | 33.6 | 38.5 | 51.1 | 76.5 | 65.2 | 56.7 |
| **Epsilonproteobacteria - Sulfurovum** | 7.4 | 13.3 | 5.5 | 4.3 | 13.0 | 28.0 | 29.2 | 19.6 | 30.0 | 31.3 | 32.7 | 45.7 | 68.6 | 63.6 | 52.4 |
| **other Epsilonproteobacteria** | 2.8 | 4.9 | 1.2 | 1.3 | 2.8 | 4.2 | 2.2 | 1.8 | 2.4 | 2.2 | 5.8 | 5.3 | 7.8 | 1.5 | 4.3 |
| **other Proteobacteria** | 4.9 | 4.4 | 7.9 | 5.4 | 2.1 | 2.4 | 1.7 | 5.3 | 3.8 | 2.2 | 3.8 | 1.1 | 0.0 | 4.5 | 6.4 |
| **other Bacteria** | 8.8 | 4.4 | 12.5 | 12.9 | 5.3 | 6.5 | 3.4 | 4.6 | 1.9 | 3.0 | 0.0 | 1.1 | 0.0 | 3.0 | 3.7 |

Suppl Table 3: Details of *K.* sp. nov ESR specimen collected for stable isotope analysis

|  | female E2 *Kiwa* | male E2 *Kiwa* | male E9 *Kiwa* |
| --- | --- | --- | --- |
| collection site | Anemone Field, n=15  chimney adjacent to Anemone field, n=5 | chimney adjacent to Anemone Field, n=8  Crab City, n=10 | Black&White, n=22  Marsh Tower, n=19  Ivory Tower, n=10 |
| carapace length in mm: mean (s.d.) [range] | 48.73 (6.79) [26-56] | 57.29 (11.37) [35-73.50] | 41.19 (9.54) [20-54] |

**References**

Hamady M, Lozupone C, Knight R (2009). Fast UniFrac: facilitating high-throughput phylogenetic analyses of microbial communities including analysis of pyrosequencing and PhyloChip data. *ISME J* **4:** 17-27.

Jackson AL, Inger R, Parnell AC, Bearhop S (2011). Comparing isotopic niche widths among and within communities: SIBER - Stable Isotope Bayesian Ellipses in R. *J Anim Ecol* **80:** 595-602.

Jackson MC, Donohue I, Jackson AL, Britton JR, Harper DM, Grey J (2012). Population-level metrics of trophic structure based on stable isotopes and their application to invasion ecology. *PLoS One* **7:** 12.

Layman CA, Arrington DA, Montana CG, Post DM (2007). Can stable isotope ratios provide for community-wide measures of trophic structure? *Ecology* **88:** 42-48.

Ludwig W, Strunk O, Westram R, Richter L, Meier H, Yadhukumar *et al* (2004). ARB: a software environment for sequence data. *Nucleic Acids Res* **32:** 1363-1371.

Parnell AC, Inger R, Bearhop S, Jackson AL (2010). Source partitioning using stable isotopes: coping with too much variation. *PLoS One* **5:** 5.

Pruesse E, Quast C, Knittel K, Fuchs BM, Ludwig W, Peplies J *et al* (2007). SILVA: a comprehensive online resource for quality checked and aligned ribosomal RNA sequence data compatible with ARB. *Nucl Acids Res* **35:** 7188-7196.

Pruesse E, Peplies J, Glöckner FO (2012). SINA: Accurate high-throughput multiple sequence alignment of ribosomal RNA genes. *Bioinformatics* **28:** 1823-1829.

Schloss PD, Westcott SL, Ryabin T, Hall JR, Hartmann M, Hollister EB *et al* (2009). Introducing mothur: open-source, platform-independent, community-supported software for describing and comparing microbial communities. *Applied and environmental microbiology* **75:** 7537-7541.

Yarza P, Richter M, Peplies J, Euzeby J, Amann R, Schleifer KH *et al* (2008). The All-Species Living Tree project: A 16S rRNA-based phylogenetic tree of all sequenced type strains. *Syst Appl Microbiol* **31:** 241-250.
